# Curriculum Innovation: A Resident-Created Multiple-Choice Question of the Week to Augment Case-Based Learning

**DOI:** 10.1212/NE9.0000000000200119

**Published:** 2024-03-18

**Authors:** Melvin Parasram, Shamelia Y. Loiseau, Andrea S. Yoo, Jacqueline B. Stone, Judy H. Ch'ang, Matthew S. Robbins

**Affiliations:** From the Department of Neurology (M.P., S.Y.L., A.S.Y., J.H.C., M.S.R.), Weill Cornell Medicine, New York; and Department of Neurology (M.P., S.Y.L., A.S.Y., J.B.S.), Memorial Sloan Kettering Cancer Center, Manhattan, NY.

## Abstract

**Introduction and Problem Statement:**

Morning report (MR) has been a foundation of learning in many neurology residency programs. However, fortification of the high-yield learning points during MR cases may be achieved with supplementary educational initiatives to promote effective long-term retention and test-enhanced learning.

**Objectives:**

During the 2020–2021 academic year, chief residents of our neurology training program sought to implement neurology certification board–style multiple-choice questions (MCQs) based on cases presented at MR to enhance case-based learning.

**Methods and Curriculum Description:**

A chief resident was selected weekly to write a MCQ based on an instructive case presented in MR from the prior week. The National Board of Medical Examiners item writing guide and online tutorial were used as guidelines for constructing MCQs. MCQs featured a clinical vignette in the question stem, and images were added to augment select cases. The MCQs were distributed using Qualtrics, which generated a web link and tracked anonymous answers. The Qualtrics link was added to the departmental weekly newsletter and labeled question of the week (QOW). Detailed explanations for each QOW were provided. A feedback survey was sent to the departmental education committee after study completion.

**Results and Assessment of Data:**

Forty MCQs were written by the chief residents, and 1 question was distributed weekly in the departmental newsletter. After week 24, the QOW was restructured to enhance visibility. The mean number of residents who completed the MCQ was 13 (of 29 neurology residents [range 4–29]). The overall median response rate was 38%. When stratified by weeks 1–24 and 25–40 to account for QOW reformatting, the median response rate for weeks 1–24 and weeks 25–40 were 24% and 55%, respectively (*p* = 0.0013). In a poststudy survey sent to the education committee, 90% of respondents felt that resident-created MCQs were similar to board-style questions and added educational value to resident learning.

**Discussion and Lessons Learned:**

A chief resident QOW initiative was feasible and led to neurology resident academic engagement and enrichment, which supplemented case-based learning through a test-enhanced learning approach. Resident participation was significantly increased with enhanced visibility of QOW in weekly emails compared with hyperlink format.

## Introduction and Problem Statement

In medical practice, a large fund of knowledge is necessary for clinical problem solving, management of patients, and clinical expertise. However, the demands and time constraints of residency training such as work hours, overwhelming volume of new information, and burnout may increase extraneous cognitive load and impair effective learning. Novel and simple educational tools can promote effective resident learning without contributing to the high demands of residency training. Morning report (MR) is a foundation of learning in many neurology residency programs. During MR, trainees discuss interesting and challenging cases with colleagues and attendings for collective learning. Fortification of the high-yield learning points during MR cases may be achieved with supplementary educational tools and initiatives to promote recall of information for effective long-term retention through test-enhanced learning.^1^ Test-enhanced learning centers on using tests to promote better retention and retrieval of information.^2^ Studies have shown that test-enhanced learning through retrieval practice in medical education is robust and a superior learning method compared with repetitive restudying.^3-5^ The effectiveness in retrieval practice is primarily due to memory consolidation and reconsolidation of learned topics via repetition of concepts and feedback.^1^ Development of an educational tool based on MR cases using concepts of test-enhanced learning theory may increase effective learning in neurology residents.

Multiple-choice questions (MCQs) of clinical vignettes are the primary question type used in medical education and on board and licensing examinations. MCQs have several advantages in medical education, such as assessment of a broad range of learner knowledge and reinforcement of learning objectives.^6^ MCQs are objective tests that measure knowledge, comprehension, application, and analysis.^7^ Furthermore, MCQs are reliable, have a high degree of validity, and are easy to administer and grade.^8^ When compared with other advance question formats such as short-answer questions and/or open-ended questions, MCQs were found to similarly assess higher-order cognitive thinking in learners.^9^ Retrieval practice with MCQs is associated with improved long-term retention of concepts.^1^ Creating MCQs with direct feedback based on presented MR cases can facilitate retrieval practice through test-enhanced learning in neurology resident education.

Well-written MCQs avoid item writing flaws and nonfunctioning distractors, include higher levels of Bloom's revised taxonomy, have higher discrimination index, and have favorable difficulty index.^10^ However, writing MCQs can be a challenging task, especially in untrained question writers. Several textbooks, journal articles, and item writing manuals are available to assist medical educators on creating effective MCQs. The National Board of Medical Examiners (NBME) provides a manual that reflects the principles of effective MCQ writing for medical education and is widely used among medical educators.^11^ Deviation from the standard MCQ writing results in poor validity and lower learner scores.^12,13^ Studies have shown that examinees have a higher educational impact from MCQs written by NBME-trained question writers compared with untrained question writers.^14^ In addition, medical students and residents who were trained on MCQ writing were found to score higher on examinations, had positively enhanced learning experiences, and were provided good engagement with clinical subject.^15-17^

MCQs based on relevant medical cases using effective test-enhanced learning model as an educational tool may increase resident learning without contributing to the high demands of residency training. During the 2020–2021 academic year, our neurology residency program sought to feasibly create board-style MCQs based on cases presented at MR as a novel education tool to augment case-based and test-enhanced learning and engagement for neurology residents.

## Objectives

The main objectives of this pilot study are listed below:

1. Outline a simple and novel learning tool that can be implemented at other residency programs.
2. Assess whether this study could be easily implemented and measure resident response rate for the QOW.
3. Assess whether better visibility of the QOW results in increased resident response rate.

## Methods and Curriculum Description

**Figure 1:**
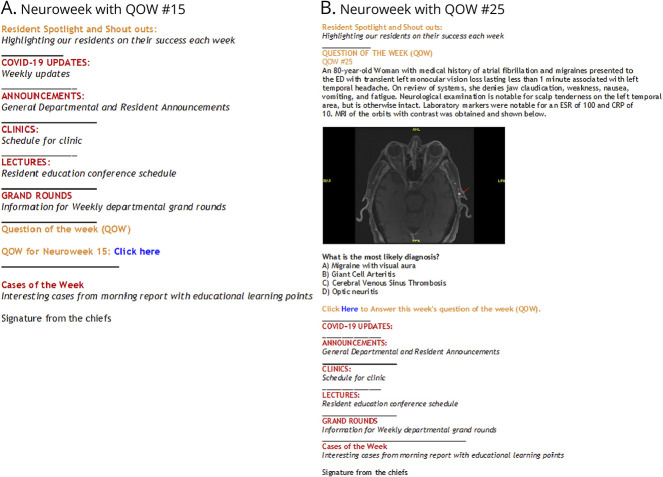
Departmental Neuroweek Emails Featuring Question of the Week The general outline for the Weill Cornell Medicine Neurology departmental weekly Neuroweek emails which include the addition of the question of the week (QOW). Figure 1A was the format used for weeks 1-24 of the study which contained the QOW as a Qualtrics hyperlink. Figure 1B was the format used for weeks 25-40 of the study, which displayed the question stem, clinical image, multiple choice question, and a Qualtrics hyperlink to submit an answer.

The chief residents (M.P., S.Y.L., and A.S.Y.) and program directors (M.S.R., J.B.S., and J.H.C.) planned to implement this education initiative before the start of the 2020–2021 academic year. A chief resident was selected weekly to write am MCQ based on an instructive case of his/her choosing, which was presented in MR from the prior week during the 2020–2021 academic year. Before initiation of this project, the chief residents were trained on MCQ writing using the NBME item writing guide and online tutorial.^11^ MCQs were designed to feature a clinical vignette, clinical images in select cases, and multiple-choice answers. Using current and relevant literature, detailed explanations for the correct and incorrect answers were instantly provided after the residents completed the MCQ for direct feedback. Citations and reference hyperlinks for explanations were also provided on the feedback page for further reading for interested residents. The MCQs were distributed using a secure Qualtrics web link to track anonymous answers. The link containing the MCQ was added to the departmental weekly newsletter and labeled question of the week (QOW) (Figure 1A). The weekly newsletter was required reading for all departmental members. As a categorical program, postgraduate year 1–4 adult neurology residents and postgraduate year 3–5 pediatric neurology residents were all invited to participate in answering the QOW (n = 29).

The QOW was conducted over 40 of 52 weeks during the 2020–2021 academic year. The QOW were not provided during the first and last 4 weeks of the academic year due to the transition period of incoming and outgoing residents and 4 weeks total during national holidays (Thanksgiving, Christmas, and New Year's Day). During week 25 of this study, the QOW was restructured to assess whether enhanced visibility would lead to an increase response rate from participating residents. Thus, weeks 1–24 contained the QOW as shown in Figure 1A (located at the bottom of the weekly emails), and weeks 25–40 contained the QOW as shown in Figure 1B (located at the top of the weekly emails).

Resident participation in the QOW was completely voluntary and confidential. For every QOW, the number of responses and correct responses were recorded anonymously through Qualtrics. The response rate and the percentage correct were calculated for each QOW. The overall median response rate and percentage correct were also calculated over the 40 weeks of the study. To assess for change in response rate after restructuring the QOW format (Figure 1), the median response rate for weeks 1–24 and 25–40 were calculated, and the percent change in median response was determined. The Mann-Whitney *U* statistical analysis was performed based on the non-normalized median response rate before and after restructuring of the QOW format.

After completion of this pilot study, a feedback survey was sent to members of the Weill Cornell Department of Neurology Education Committee, which comprised 17 faculty serving in administrative education roles. The education committee included 2 members who participated in writing American Board of Psychiatry and Neurology (ABPN) board–style questions for organizations outside Weill Cornell Department of Neurology, an associate dean of medical education at Weill Cornell School of Medicine, and 2 members who hold advanced degrees in education. The feedback survey was optional, and responses were anonymous. Survey questions are provided in the supplementary data section. The feedback survey was sent to the education committee members to obtain their evaluation and feedback on the pilot study structure, evaluation of resident-created MCQs when compared with ABPN board–style questions, and potential impact of this study on neurology resident education.

### Standard Protocol Approvals, Registrations, and Patient Consents

The design of this project was submitted to the Weill Cornell Medicine Institutional Review Board (IRB) for review (Protocol 21-04023521) and was found to be exempt because the project qualified as not human subject research. As such, the IRB did not need to monitor or review personnel changes regarding this study because it did not fall under their purview because it is not considered human subject research as defined under Health and Human Services Regulation 45 CFR 46.102 (f). Resident participation was optional, and consent for residents to participate in the study was implied by answering the QOWs.

### Data Availability

Anonymized data not published within this article will be made available by request from any qualified investigator.

## Results and Assessment Data

During the 2020–2021 academic year, a total of 40 MCQs were written by the chief residents, and 1 question was distributed weekly in the departmental newsletter email. The question topics for the QOW included neuroimmunology (9, 22.5%), pediatric neurology (7, 17.5%), neuromuscular disorders (4, 10%), headache medicine (4, 10%), neurocritical care (3, 7.5%), neuro-oncology (3, 7.5%), cognitive neurology (3, 7.5%), vascular neurology (2, 5%), neuroinfectious disease (2, 5%), epilepsy (2, 5%), and neuro-ophthalmology (1, 2.5%) (eTable 1).

**Figure 2:**
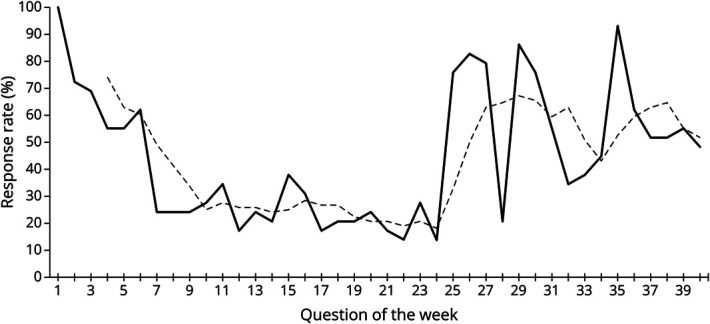
Resident Response Rate to the Question of the Week This line graph shows the response rate of neurology residents for each question of the week (QOW). The solid line represents the response rate for each QOW and the dashed line represents the 4-week average response rate trendline. There was a steady decline the response rate per week and the 4-week average response rate until week 24. The format of the QOW was changed after week 24, which was associated with a subsequent increase in the response rate per week and when averaged over 4 weeks until week 40.

The mean number of residents who completed the MCQ each week was 13 (range 4–29) (eTable 1). The overall median response rate and percentage correct were 38% and 71%, respectively (Figure 2). When stratified by weeks 1–24 and 25–40 to account for QOW reformatting, the median response rate for weeks 1–24 and weeks 25–40 were 24% and 55%, respectively (*p* = 0.0013, Mann-Whitney *U* test). The median response rate increased by 129% after week 24.

After completion of the study, an anonymous and voluntary 5-question feedback survey was sent to the Weill Cornell Department of Neurology Education Committee (eFigure 1). Ten members of the committee completed the survey (response rate of 58%). When committee members were asked whether the QOW format was easy to use and navigate, 50% strongly agreed, 20% somewhat agreed, and 30% neither agreed nor disagreed (eFigure 2, question 1). For feedback questions asking whether the QOW was similar to the ABPN board-style MCQs, whether the QOW added educational value to neurology resident learning, and whether the QOW should continue in subsequent academic years to increase resident academic engagement and educational enrichment, 50% strongly agreed, 40% somewhat agreed, and 10% neither agreed nor disagreed for each question (eFigure 2, questions 2–4). Fifty percent strongly agreed, 40% somewhat agreed, and 10% somewhat disagreed that the QOW may have a positive impact on residency in-service training examination (RITE) scores (eFigure 2, question 5).

## Discussion and Lessons Learned

The goal of this pilot study was to assess the implementation of resident-created multiple-choice board-style questions to enhance neurology resident learning experience based on cases presented at MR during the 2020–2021 academic year. During the 40 weeks of this study, the most common question topics written for the QOW were neuroimmunology and pediatric neurology. The overall median resident response rate for this study was 38%. While there is no standard consensus on acceptable optional survey response rates in the medical literature, studies have found average response rates for medical surveys ranging from 27% to 75%.^18-24^ Resident participation and response rate in this study were comparable with average response rates in optional medical surveys. Therefore, the median response rate for this study is considered to reflect good resident engagement. The median percentage correct was 71% for the QOW over 40 weeks of this study. In a study assessing the association of the neurology RITE scores with ABPN board examination pass rates, the authors found a RITE score average of 60%–67% among residents in 2008 and 2009 and found a positive correlation with ABPN passing rates.^25^ The median percentage correct in our study was consistent with the average RITE score, which may reflect an appropriate level of difficulty of our QOW initiative.

There was a steady decline in resident response rate in the first 24 weeks of this study. The format of the QOW was changed to enhance visibility in the departmental emails for weeks 25–40. Before reformatting the QOW, the median resident response rate was 24%, and there was a statistically significant increase in median resident response rate to 55% after reformatting the QOW. This indicates that visualization of the QOW increased resident interest, participation, and response rates. This visualization effect is seen in medical journals in which visual abstracts widely disseminate information, increase readability and engagement, and increase Altmetric scores of the journal article compared with written abstract alone.^26-28^ Further optimizing visual presentations of MCQs may increase resident engagement and response rates, which can lead to enrichment of residents learning.

In our study, residents learned about interesting neurologic cases during MR, and select cases were subsequently tested with the QOW, which demonstrates effective use of test-enhanced learning to enhance learner retention and retrieval of learned concepts for resident medical education. For tests to be effective memory-enhancing interventions, the subject matter should be directly tested soon after the learning experience.^29^ The QOW was available within 1 week of a selected case presentation at MR, which allowed for immediate retention of learned concepts. Direct feedback was provided for each QOW, which augments the test-enhanced learning model by teaching the learner the reasoning for the correct answer, fortifying learned concepts, and increasing retention of learned concepts.^30^ In addition, the QOW was administered frequently throughout the academic year. Several studies have demonstrated that repeated testing has shown to increase retention of subject matter than taking a single test.^4,31^ The authors believe that the study design implemented test-enhanced learning to our residents and this framework can be used in other residency programs.

This resident-led educational intervention may have facilitated situated learning for the QOW writers and participants. The situated learning theory emphasizes that learning is best acquired through active participation and social interaction within a community of practice.^32,33^ Residents collectively learned about interesting cases during MR among a community of expert attending neurologists and chief residents who worked together to create novel MCQs based on these cases for the QOW. Situated learning occurred with resident participants because the QOW reflected real-life clinical scenarios and the resident participant had to use critical thinking to answer the clinical question. The benefit of situated learning includes knowledge attainment and appropriate application of learned concepts in real-life context.^32,34^ In addition, microlearning, in the form of weekly, short, single MCQs, may have occurred among resident participants. Microlearning reduces learner fatigue and has a positive effect on learner retention and engagement in collaborative learning.^35,36^

This study has several limitations. One important consideration for this study was the educational quality of the MCQs and detailed answer explanations written by the chief residents. The authors attempted to minimize poorly written MCQs by having the chief residents undergo self-guided question-writing training using the NBME item writing guide and online tutorial.^11^ Prior studies have shown high-quality MCQs from NBME-trained question writers compared with those from untrained question writers.^14^ The discrimination index for each QOW could not be performed given the anonymization of resident responses, which limits measuring the quality and reliability of MCQs. As an indirect measure of the quality and validity of MCQs, a poststudy feedback survey was sent to the neurology department education committee, which consisted of a cohort of medical education experts. In this poststudy feedback survey, education committee members were specifically asked whether the resident-created MCQs were similar to ABPN board–style MCQs (eFigure 2, question 2). All education committee members viewed the QOWs because the weekly departmental newsletter email were considered required reading by all staff members. Approximately 90% of respondents from the education committee agreed that the QOWs were similar to the ABPN questions. This may suggest that the quality of the resident-written MCQs were of equivalent quality to board-style MCQs. However, this assessment may be limited because it may not provide sufficient quality of evidence that the resident-created QOWs were equivalent to ABPN questions. In addition, the education committee cohort consisted 5 of 17 members who are experts in MCQ writing and medical education. Outside the feedback survey to the departmental education committee, there were no external assessments of the chief resident–created MCQs, which may weaken the external validity of this study.

Though our study was a pilot to assess initial implementation at a single institution, another limitation was lack of direct outcomes from residents who regularly engaged in QOW and assessment of the possible benefits of test-enhanced learning in medical education. The anonymity of responses did not permit us to directly measure outcomes such as improved knowledge, RITE scores, and ABPN passing rates. In addition, the anonymity of responses did not allow for stratification of respondents into level of postgraduate training to assess and compare differences in participation and study results. Last, the amount of MCQs during the academic year may be questioned. The disadvantage of 40 MCQs is that the low quantity may limit the strength of this study. However, because the goal was initial implementation of this project and responses from residents were optional, the authors believed that more than 1 MCQ per week may preclude resident participation given the challenging demands of residency training. Thus, the authors believe that 1 MCQ per week lead to the successful implementation of this study and resident engagement and microlearning without contributing to burnout during training.

Our study shows that resident-created multiple-choice QOW led to good neurology resident academic engagement and educational enrichment by supplementing case-based learning using a test-enhanced learning model. The authors assessed resident engagement as a proxy for feedback. As a pilot study, we sought feedback from education experts in the departmental education committee. Approximately 90% of respondents from the neurology department educational committee agreed that this study added educational value to neurology resident learning and should continue in subsequent academic years and 70% of respondents agreed that the format of the QOW was easy to use in a poststudy survey. This is a novel study that incorporated resident-created MCQs based on real cases presented at MR to enhance resident learning in neurology.

Future directions may include optimizing MCQ visibility and delivery strategies to increase resident participation, additional methods of objective and external evaluation of resident-created MCQs, assessment of the long-term impact of the QOW on resident performance on the RITE and the ABPN board examination, poststudy questionnaire for participating residents to gain insight into their experience with this study using test-enhanced learning concepts and additional feedback to improve this educational tool, and assessment of potential benefits and impact of test-enhanced learning in case-based learning and in medical education. Furthermore, this educational initiative could be used in other residency and fellowship programs to increase trainee learning experience.

A pilot study involving the implementation of a chief resident-created multiple-choice QOW based on MR cases using test-enhanced learning was performed with good resident participation and response rate. Resident participation was significantly increased with enhanced visibility of MCQs in weekly emails compared with the hyperlink format. The data obtained and feedback from the educational committee suggest that this study led to neurology resident academic engagement and enrichment, which supplemented case-based learning. Future directions that can strengthen this study include collection of data in a way to allow for calculation of discrimination index of questions and assessment of long-term impact of the QOW on resident performance on the RITE and ABPN board examination.

## Appendix Authors

**Table:**

Name	Location	Contribution
Melvin Parasram, DO, MS	Department of Neurology, Weill Cornell Medicine, New York; Department of Neurology, Memorial Sloan Kettering Cancer Center, Manhattan, NY	Drafting/revision of the article for content, including medical writing for content; major role in the acquisition of data; study concept or design; and analysis or interpretation of data
Shamelia Y. Loiseau, MD	Department of Neurology, Weill Cornell Medicine, New York; Department of Neurology, Memorial Sloan Kettering Cancer Center, Manhattan, NY	Drafting/revision of the article for content, including medical writing for content; major role in the acquisition of data; and analysis or interpretation of data
Andrea S. Yoo, MD	Department of Neurology, Weill Cornell Medicine, New York; Department of Neurology, Memorial Sloan Kettering Cancer Center, Manhattan, NY	Drafting/revision of the article for content, including medical writing for content; major role in the acquisition of data; and analysis or interpretation of data
Jacqueline B. Stone, MD	Department of Neurology, Memorial Sloan Kettering Cancer Center, Manhattan, NY	Drafting/revision of the article for content, including medical writing for content
Judy H. Ch'ang, MD	Department of Neurology, Weill Cornell Medicine, New York, NY	Drafting/revision of the article for content, including medical writing for content
Matthew S. Robbins, MD	Department of Neurology, Weill Cornell Medicine, New York, NY	Drafting/revision of the article for content, including medical writing for content; study concept or design; and analysis or interpretation of data

## Glossary

ABPN: American Board of Psychiatry and Neurology
IRB: institutional review board
MCQ: multiple-choice question
MR: morning report
NBME: National Board of Medical Examiners
QOW: question of the week
RITE: residency in-service training examination
